# The safety and efficacy of oral immunotherapy compared to epicutaneous immunotherapy in peanut allergen desensitisation amongst the paediatric cohort—a narrative review

**DOI:** 10.3389/falgy.2025.1613237

**Published:** 2025-05-20

**Authors:** Ehtesam A. Chowdhury, Olivia C. Jadeja

**Affiliations:** ^1^ Department of General Surgery, Hereford County Hospital, Wye Valley NHS Trust, Hereford, United Kingdom; ^2^ Department of General Surgery, Southmead Hospital, North Bristol NHS Trust, Bristol, United Kingdom

**Keywords:** peanut allergen, immunotherapy, desensitisation, oral immunotherapy (OIT), epicutaneous immunotherapy (EPIT)

## Abstract

Peanut allergies result from a type 1 hypersensitivity reaction, with a prevalence of approximately 1% in children under 5 years of age. The allergens that instigate this reaction are the peanut proteins (Ara h 1–Ara h 8) for which IgE antibodies are specifically produced. Allergen immunotherapy (AIT), despite the uncertainty regarding its mode of action, has been increasingly utilised with the aim of desensitisation against these allergens. AIT encompasses various modes of administration, including epicutaneous immunotherapy (EPIT) and oral immunotherapy (OIT). The review adheres to Preferred Reporting Items for Systematic reviews and Meta-Analyses guidelines, with a comprehensive literature search conducted using databases including MEDLINE®, Embase™, PubMed®, and Google Scholar™. Search terms targeted OIT and EPIT in the desensitisation and management of peanut allergy in children, with studies spanning the past 20 years included based on predefined eligibility criteria. The extent of the immunotherapies' efficacy and safety in children is yet to be thoroughly established; however, OIT demonstrated increased desensitisation rates amongst children when compared to EPIT. The long-term efficacy has not been fully established, with sustained unresponsiveness not reported within most studies. Both modes of administration had a high proportion of participants experiencing adverse effects (AEs), with gastrointestinal symptoms more common with OIT and cutaneous reactions with EPIT. Serious AEs were observed less frequently, however, systemic reactions such as anaphylaxis were more apparent with OIT. Future research should focus on peanut EPIT, as the literature was relatively scarce. Furthermore, research studies should assess sustained unresponsiveness to fully gauge the long-term effects of AIT in children.

## Introduction

Peanut allergy, affecting approximately 1% of all children under the age of 5, is caused by a type 1 hypersensitivity reaction mediated by immunoglobulin (Ig) E antibodies (1). These allergens are the proteins within the peanuts, ranging from Ara h 1 to Ara h 8. In predisposed individuals, IgE antibodies are produced specifically for these proteins in the sensitisation phase of the reaction (2). IgE antibodies specific to Ara h 1 and Ara h 2 are most prevalent, occurring in over 90% of individuals allergic to peanuts (3). In the effector phase, these synthesised IgE bind onto mast cells and basophils, using the high-affinity receptor, Fc*ε*RI (4). Upon subsequent exposure to these proteins, cross-linking of IgE antibodies occurs. This elicits mast cells and basophil degranulation, releasing many inflammatory mediators such as prostaglandins, histamines, leukotrienes, platelet-activating factor, and many more. This leads to pruritus, erythema, oedema, and systemic reactions such as anaphylaxis, which can lead to airway, breathing, and circulatory compromise (5).

Historically, the main therapeutic modality of peanut allergy has been through strict avoidance of the allergen (6). However, to date, allergen immunotherapy (AIT) has been an increasingly emergent therapy for food allergies (7). This is evident with the novel approval of Palforzia, a type of AIT licensed for peanut allergy in children (8). The administration of AIT has three main types: oral immunotherapy (OIT), sublingual immunotherapy (SIT), and epicutaneous immunotherapy (EPIT) (9). Their mechanism, whilst still relatively unclear, is thought to be through gradual low-dose exposure to the allergen, leading to desensitisation of the effector cells (mast cells and basophils) (10). In addition, immunotherapy has been shown to modulate the immune response through skewing the balance from a pro-allergic T_H_2 response (decreasing the amount of secreted inflammatory mediators) to an increased T_H_1 regulatory environment. Furthermore, it also affects the T_REG_ cellular response by secreting interleukin 10 (IL-10) and transforming growth factor beta (TGFβ), thus impairing IgE production and inducing synthesis of blocking-type IgG4 in turn, initiating peripheral tolerance (9). This review aims to compare the effectiveness of OIT with EPIT for peanut allergies in children and to assess the safety of each in the paediatric cohort.

## Methods

This review ensured that the Preferred Reporting Items for Systematic reviews and Meta-Analyses (PRISMA) guidelines were followed throughout.

The MEDLINE®, Embase™, PubMed®, and Google Scholar™ databases were searched for suitable studies. A combination of Boolean operators was utilised to facilitate search sensitivity. In addition, with the usage of the patient, intervention, comparison, and outcome (PICO) framework, the search was further refined to meet the objectives of this narrative review. The search terms were (“oral immunotherapy”) AND (“epicutaneous immunotherapy” OR “skin patch”) AND (“peanut allergy” OR “peanut allergen”) AND (“children” OR “paediatric”) AND (“desensitis(z)ation”) AND (“safety” OR “efficacy” OR “outcomes”).

Limits included original research articles written in English within the past 20 years.

This review did not require ethical approval as it involved examining previously published literature.

Articles involving the paediatric cohort were considered. The focus of this review was to understand and compare these different modes of immunotherapy in the desensitisation of peanut allergy within this cohort. Consequently, studies that analysed efficacy and commented on safety outcomes were included. Furthermore, the studies were examined based on the similarity of the methodology used, namely, using an oral food challenge (OFC), as this would allow for a more enhanced comparison. Full details of the inclusion and exclusion criteria of study selection are provided in Table 1. After having reviewed the literature, six main articles were chosen for this review.

**Table 1:** Inclusion and exclusion criteria for article eligibility.

Criterion	Inclusion	Exclusion
Research focus	Studies focusing on immunotherapy for peanut allergy	Research relating to other allergic pathologies
Participants	Studies evaluating the paediatric cohort based on their local definitions of this population	Any study solely evaluating the non-paediatric population
Type of study	Original and primary research	Commentaries, abstracts, conference abstracts, and letters to the editor
Methodology	Quantitative/qualitative/mixed methodology	
Timescale	Studies published between 2025 and 2005 (past 20 years)	Studies published before 2005

## Results and discussion

Results for all studies used are represented in Table 2.

**Table 2:** The results of the main studies analysed in this review.

Study	Mode of immunotherapy	Primary outcome met (%)	Statistical results
Fleischer et al. (11)	Epicutaneous	35.3 with peanut 250 μg patch treatment 13.6 with placebo patch treatment	Difference, 21.7 (95% CI, 12.4–29.8; *p* < 0.001)
Kunnel and Varshney (12)	Epicutaneous	46 with 100 μg patch treatment 48 with 250 μg patch treatment 12 with placebo patch treatment	*p* = 0.005 for 100 μg patch treatment compared with placebo *p* = 0.003 for 250 μg patch treatment compared with placebo
Zhong et al. (14)	Oral	67 at the 6-month oral food challenge 14 with sustained unresponsiveness	N/A
Simons (18)	Oral	62 in the active group 0 in the control group	95% CI, 45–78 in the active group 95% Cl, 0–9 in the control group *p* < 0.001
Blumchen et al. (13)	Oral	74.2 in the active group 16.1 in the placebo group	*p* < 0.001
Chipps (15)	Oral	67.2 in the active group 4 in the placebo group	Difference, 63.2 (95% CI, 53.0–73.3, *p* < 0.001)

The mode of immunotherapy used is indicated for each study alongside quantitative results/data relating to each of their primary outcomes. Primary outcomes were mainly classified as participant success with their respective treatments. Statistical data have been included where available.

### Treatment efficacy of EPIT and OIT

Two studies focusing on EPIT by Fleischer et al. (11) and Kunnel and Varshney (12) arrived at conclusions that peanut EPIT was, to an extent, efficacious in eliciting desensitisation to ingested peanut protein. Within the study conducted by Fleischer et al. (11), treatment success was defined as a person responding to the treatment. This was further specified by either having a baseline OFC peanut allergy symptom-eliciting dose of <10 mg with a 12-month OFC peanut allergy symptom-eliciting dose of ≥300 mg, or a baseline OFC peanut allergy symptom-eliciting dose of 10–300 mg with a 12-month OFC peanut allergy symptom-eliciting dose of >1,000 mg. In contrast, the treatment success criteria adopted by Kunnel and Varshney (12) required participants to ingest ≥5,044 mg in an OFC at month 12, or a 10-fold increase in peanut protein ingestion from baseline to an OFC at month 12.

The two studies demonstrated statistically significant results when compared to their respective placebo groups, however, the study by Kunnel and Varshney (12) revealed a greater proportion of efficacious results in both of their active groups (100 and 250 μg peanut protein patches) compared to the placebo. The size of the participant population was significantly smaller in the study by Kunnel and Varshney (12) (with a difference of 282, not including discontinuations), which could have skewed the results, disallowing for generalisability. Furthermore, the inclusion criteria differed subtly between the two studies with Fleischer et al. (11) utilising a minimum peanut skin prick test (SPT) wheal size of ≥6 mm and a peanut specific IgE level of >0.7 kUA/L compared with a peanut SPT wheal size of ≥3 mm and peanut specific IgE level of >0.35 kUA/L. This suggests that the participants in the study conducted by Fleischer et al. (11) were more sensitive to peanut protein allergens and hence may have required a longer duration of EPIT to show more efficacious results.

Both EPIT studies included paediatric participants, but only Fleischer et al. (11) included them exclusively with a participant age range of 4–11 years. Although Kunnel and Varshney (12) included participants in the age range of 4–25 years, they observed a higher treatment success rate amongst those who were 4–11 years vs. greater than 11 years (*p* = 0.03). This may have been attributed to the absorption rates of the epicutaneous patches, as children tend to have a larger surface area to volume ratio when compared to adolescents and adults, thereby affecting absorption. This reinforces the idea that EPIT is more efficacious in children.

To investigate the efficacy of OIT, four studies were selected. All the studies had primary efficacy outcomes similar to those of the EPIT studies, which included desensitisation or being a treatment responder. Within these four, the range of treatment success was 12.2%, with the study by Blumchen et al. (13) exhibiting the highest efficacy of 74.2%. The number of participants included in these studies varied significantly, ranging from 487 to Zhong et al. (14) with only 9. This small sample size limits the statistical power of the study, making it more difficult to detect true effects and increasing the risk of random error. A small range between treatment efficacy results and a large range in participant populations suggests that OIT is able to translate its success without group size being a significant factor, although to confirm this, a thorough statistical analysis would need to be conducted to comment on this significance. Moreover, when comparing active treatment groups to the placebo (or control) in all of the studies, there was a statistical significance with *p* < 0.001 (where applicable). This, along with the conclusion by Chipps (15) stating that there was no significance in the efficacy of OIT for the participants aged 18–55, further highlights the efficacious nature of OIT in children.

Compared to the EPIT studies, OIT displayed an overall increased treatment success rate and, hence, increased efficacy. A contributing factor could be the mode of administration of the therapy. With EPIT, patches containing low doses of peanut protein were used, however, in the OIT studies, flour containing the appropriate peanut protein dosage was mainly given to participants, with the exception of Chipps (15), who used a peanut-derived biological OIT drug. Rates of absorption, therefore, could have played a part with increased rates of absorption favouring oral administration (16). Furthermore, a lack of proper adherence to the therapy may have played a role in this. With EPIT, adherence appeared stricter as patches were applied on the interscapular space, ensuring that they did not peel off. Any rigorous movements, such as those in sports, may have compromised the results due to the underabsorption of the correct doses. Moreover, participants who may have experienced this were more likely to underreport it as an adherence issue, as it would have been a direct result of the participants’ actions rather than a patch issue. It is important to keep in mind that the definition of treatment success reported across studies differed, which, although similar, introduces a risk of inconsistency in outcome interpretation. This could potentially limit comparability in the overall findings.

In addition, sustained unresponsiveness (SU) to peanut allergens was only monitored in the study by Zhong et al. (14). Only one participant tolerated 6,000 mg peanut protein again compared to the initial OFC, with the rest of those undergoing abstinence periods showing a reduced tolerance of more than 38%. This shows that, although efficacious, desensitisation was rather short-lived. Future research with participants undergoing an abstinence period would be needed to further comment on the efficacy.

### Treatment safety of EPIT and OIT

Adverse effects (AEs) were a common theme in all the studies in both the active and placebo groups, with the majority of participants experiencing at least one AE. The nature of these AEs differed between EPIT and OIT. Local cutaneous reactions occurred more frequently with the use of EPIT, which were mainly limited to the treatment patch site. This was emphasised by Kunnel and Varshney (12) reporting a maximum of 0.2% of doses given to participants, in any of the treatment groups, resulting in a non-patch site reaction. Gastrointestinal tract (GIT) symptoms, such as abdominal pain, was almost exclusively observed in the studies relating to OIT, with Fleischer et al. (11) highlighting that there was no AE attributable to gastrointestinal concerns, unlike Chipps (15), who reported that such an AE occurred in 85.8% of the active treatment participants. Nevertheless, both modes of administration of AIT demonstrated a low number of participants experiencing serious adverse effects (SAEs).

Systemic reactions, such as anaphylaxis, were only observed in the EPIT study by Fleischer et al. (11) and the OIT study by Chipps (15). Chipps (15) reported that systemic allergic reactions occurred in 14.2% of the participants, with 60 of them requiring intramuscular epinephrine (14% of the active treatment group). In contrast, 6.4% of participants experienced systemic allergic reactions in the EPIT study, with 3.4% of treatment patch participants experiencing 10 episodes of anaphylaxis related to the treatment. In total, 40% of these episodes were treated with intramuscular adrenaline. This suggests an increased risk of systemic reactions with the use of OIT and is further reinforced by the systematic review by Chu et al. (17), who came to a similar conclusion. On the contrary, EPIT demonstrated standardisation across doses administered to participants through the use of 250 μg cutaneous patches. The studies involving OIT used varying doses. The lack of this standardisation may also contribute to this trend seen with OIT and systemic reactions.

## Conclusion

Overall, OIT showed increased efficacy, to an extent, among children compared to its epicutaneous counterpart. However, OIT resulted in higher rates of SAEs, namely through systemic reactions such as anaphylaxis, suggesting it is a riskier approach to AIT than the epicutaneous mode of administration. With the recent approval of OIT in the form of Palforzia, established data regarding this treatment could prove vital in assessing and comparing this mode of AIT. In the future, more paediatric *in vivo* research is required, especially in EPIT due to the scarcity of studies, to assess the extent of the desensitisation by observing sustained unresponsiveness.
